# Common Variation in *EDN1* Regulatory Regions Highlights the Role of PPARγ as a Key Regulator of Endothelin *in vitro*

**DOI:** 10.3389/fcvm.2022.823133

**Published:** 2022-02-25

**Authors:** Mauro Lago-Docampo, Carlos Solarat, Luis Méndez-Martínez, Adolfo Baloira, Diana Valverde

**Affiliations:** ^1^CINBIO, Universidade de Vigo, Vigo, Spain; ^2^Rare Diseases and Pediatric Medicine, Galicia Sur Health Research Institute (IIS Galicia Sur), SERGAS-UVIGO, Vigo, Spain; ^3^Department of Biotechnology and Aquaculture, Institute of Marine Research (IIM-CSIC), Vigo, Spain; ^4^Pneumology Department, Complexo Hospitalario Universitario de Pontevedra, Pontevedra, Spain

**Keywords:** Endothelin-1 (ET-1), PPARγ (peroxisome proliferator-activated receptor gamma), KLF4, VDR (vitamin D receptor), UTR-untranslated regions, common variation, *EDN1* mRNA

## Abstract

Pulmonary Arterial Hypertension (PAH) is a rare disease caused by the obliteration of the pulmonary arterioles, increasing pulmonary vascular resistance and eventually causing right heart failure. Endothelin-1 (EDN1) is a vasoconstrictor peptide whose levels are indicators of disease progression and its pathway is one of the most common targeted by current treatments. We sequenced the EDN1 untranslated regions of a small subset of patients with PAH, predicted the effect *in silico*, and used a luciferase assay with the different genotypes to analyze its influence on gene expression. Finally, we used siRNAs against the major transcription factors (TFs) predicted for these regions [peroxisome proliferator-activated receptor γ (PPARγ), Krüppel-Like Factor 4 (KLF4), and vitamin D receptor (VDR)] to assess EDN1 expression in cell culture and validate the binding sites. First, we detected a single nucleotide polymorphism (SNP) in the 5' untranslated region (UTR; rs397751713) and another in the 3'regulatory region (rs2859338) that altered luciferase activity *in vitro* depending on their genotype. We determined *in silico* that KLF4/PPARγ could bind to the rs397751713 and VDR to rs2859338. By using siRNAs and luciferase assays, we determined that PPARγ binds differentially to rs397751713. PPARγ and VDR Knock-Down (KD) increased the *EDN1* mRNA levels and EDN1 production in porcine aortic endothelial cells (PAECs), while PPARγ and KLF4 KD increased the EDN1 production in HeLa. In conclusion, common variants in EDN1 regulatory regions could alter EDN1 levels. We were able to validate that PPARγ binds in rs397751713 and is a key regulator of EDN1. In addition, KLF4 and VDR regulate EDN1 production in a cell-dependent manner, but VDR does not bind directly to the regions we studied.

## Introduction

Pulmonary Arterial Hypertension (PAH) is a rare and devastating disease that involves the thickening of the pulmonary arterioles, leading to an increase in pulmonary vascular resistance and eventually right heart failure (1). The pathophysiology of PAH starts with the remodeling of the arteries (2), where there is an accumulation of different cell types (endothelial, smooth muscle, fibroblasts, and pericytes) (2). This is coupled with increased inflammation and immune cell infiltration to obliterate the precapillary arteries (3). The genetic basis of PAH has been slowly uncovered during the past decades, where more than 16 genes involved in PAH have been reported with different degrees of evidence (4). Some genes may modulate the evolution of the disease. The *Endothelin-1* gene (*EDN1*) is one of them. The effects of this peptide are widely implicated in PAH and its treatment. Endothelin receptor antagonists (ERAs) are used to treat the progression of PAH (5).

The family of endothelins (ET) is constituted by three isoforms of 21 amino acids (6). ET-1 is the predominant isoform and the most important by its biological function. It is an endogenous and strong vasopressor synthesized mainly by the vascular endothelium. Most ET-1 is released toward the smooth muscle cells in a paracrine/autocrine way, and pulmonary circulation is its main clearance site (7). However, it can be detected in plasma or serum.

The disruption of the balance between vasoconstriction and vasodilation triggers a wide variety of pathologies in different organs and tissues. Pulmonary vessels are one of the main targets of ET-1. Plasma levels of ET-1 have been correlated with the severity of PAH and its prognosis, in particular, when associated with the plasma levels of ET-3, because of this, the ET-1/ET-3 ratio has proven to be a powerful PAH prognostic indicator (8–10). The enhanced activity of the endothelin system has been implicated in PAH severity for a long time (11), despite the fact that the amount of experimental data demonstrating this is still limited (12).

In this work, we looked for variants in the UTR regions of *EDN1* to analyze its influence on gene regulation.

## Materials and Methods

### Cohort Description

The cohort was composed of 36 patients of group I PH: 21 IPAH and 15 Associated PAH (10 connective tissue disease, three HIV, and two congenital heart disease). The IPAH group was composed of 15 women and six men (71% women; 29% men). While in the associated forms, seven were women and eight men (47% women; 53% men). The mean age at diagnosis was 56 ± 16. Most of the patients had been described in previous studies from our group (13). The study was approved by the ethical committee for scientific research of Galicia (*Comité Ético de Investigación Cl*í*nica de Galicia*) and followed the clinical-ethical practices of the Spanish Government and the Helsinki Declaration.

### Mutational Screening

We extracted the genomic DNA from peripheral blood mononuclear cells using the FlexiGene DNA Kit (Qiagen, Hilden, Germany) according to the instructions of the manufacturer. DNA amplification was performed with 50 ng of genomic DNA from each individual by PCR using the NZYtaq II Green Master Mix (NZYtech, Caparica, Portugal). The primers used for each of the EDN1 gene regions are described in Supplementary Table S1. The amplification conditions were 95°C for 2 min, 35 cycles of 1 min at 95°C, 30 s at each couple of primers annealing temperature (Supplementary Table S1), and 1 min at 72°C; followed by 5 min at 72°C. PCR products were separated by electrophoresis through 1 or 2% agarose gels stained with Ethidium Bromide. To confirm fragment length, Brightmax 500-10 Kb DNA Ladder (Canvax, Córdoba, Spain) and NZYDNA Ladder V (NZYtech, Caparica, Portugal) were used as molecular weight markers. PCR fragments were purified using ExoSAP-IT kit (ThermoFisher, Waltham, USA), and sequencing was carried out in the C*entro de Apoio Cient*í*fico-Técnico á Investigación* (CACTI) of the Universidade de Vigo. Every sample was sequenced independently in both forward and reverse strands to confirm the results obtained. Lastly, sequences were aligned to the reference ENSEMBL DNA sequence [ENST00000379375.5].

### *In silico* Effect and Conservation Analyses

Transcription factor (TF) candidates were determined using the online software MatInspector (Genomatix, Germany) (14). This bioinformatics software provides information on TFs capable of binding to the genomic regions analyzed. We also looked for regulatory motifs and *EDN1* conservation using the University of California Santa Cruz (UCSC) Genome Browser (15).

### Design of the Luciferase Constructs

To analyze the 5' untranslated region (UTR) variants, we used the pGL3-Basic Luciferase Reporter Vector (Promega, Madison, USA), amplification of the 1.4 kb of the 5' UTR region was carried out using Phusion High Fidelity Polymerase (ThermoFisher, Waltham, USA) with the primers shown in Supplementary Table S2. Fragment and plasmid were digested with NheI and XhoI (NZYtech, Caparica, Portugal), ligated with T4 ligase (Canvax, Córdoba, Spain), and used to transform NZYstar competent cells (NZYtech, Caparica, Portugal). The empty pGL3-Basic was used as a negative control, and pRL-CMV (Promega, Madison, USA) was used as an internal control.

The 3' variants were transfected using the pmirGLO Dual-Luciferase vector (Promega, Madison, USA). A fragment of 1.4 kb was amplified and cloned as stated above using SalI instead of XhoI. Empty vector was used as the positive control.

### Cell Culture and Transfection

HeLa cells (ATCC: CCL-2) were cultured in Dulbecco's Modified Eagle Medium (DMEM; ThermoFisher, Waltham, USA) supplemented with 10% Fetal Bovine Serum (FBS) (ThermoFisher, Waltham, USA), 1% streptomycin/penicillin (Lonza, Basel, Switzerland), at 37°C with 5% CO_2_ and humidified atmosphere. Porcine aortic endothelial cells (PAECs; Merck: 302-05A, ECACC, Porton Down, UK) were cultured in Endothelial Cell Growth medium (Sigma-Aldrich, San Luis, USA) supplemented with 10% FBS and 1% streptomycin/penicillin. PAECs were used between passages three and seven.

For the luciferase assay, we plated 40,000 HeLa cells per well in 24-well plates, at least four replicates per condition were used and analyzed on different days. When the cells showed 80–90% confluence, transfection was carried out using 0.5 μg per well of plasmid DNA and Lipofectamine 2,000 (ThermoFisher, Waltham, USA) in a 1:3 reagent: DNA ratio, following the instructions of the manufacturer. The pGL3 plasmid was co-transfected with 20 ng of pRL-CMV to allow normalization against *Renilla* luciferase.

For immunofluorescence, we seeded 15,000 HeLa/PAECs per well in μ-slide 8-well chambers (ibidi, Gräfelfing, Germany), 24 h later we proceeded with transfection.

We performed the Knock-Down (KD) of Krüppel-Like Factor 4 (KLF4), peroxisome proliferator-activated receptor γ (PPARγ), and Vitamin D receptor (VDR) using a commercial pool of small interfering RNAs (siRNAs) (Dharmacon, Lafayette, USA) at a concentration of 100 nM for both PAEC and HeLa cells, transfection was carried out with Lipofectamine RNAiMax (ThermoFisher, Waltham, USA) following the instructions of the manufacturer, 24 h after the transfection, we changed the media, and 24 h later, cells were harvested to assess KD efficiency and *EDN1* mRNA levels.

### Luciferase Assay

We transfected the siRNAs in HeLa cells for 24 h, then we changed the media, and 24 h later, we transfected the cells with the different luciferase constructs depending on the target gene (pGL3-ET-1prom for *KLF4* and *PPAR*γ, and pmirGLO-EDN1 for *VDR*). We then proceeded with a conventional luciferase assay as described briefly below.

Cells were harvested 36 h post-transfection. The assay was performed using the Dual-Glo Luciferase system (Promega, Madison, USA) following the instructions of the manufacturer, the assay was read in ½ area 96-well white plates (Corning, New York, USA) on an EnVision 2104 (Perkin Elmer, Waltham, USA).

Data were normalized using the firefly/renilla ratio and then scaled to the most common genotype (for the triple genotype comparison) or the empty vector (for the TF binding site test).

### Quantitative(q) PCR

RNA extraction was carried out using NZY Total RNA Isolation Kit (Nzytech, Caparica, Portugal) following the instructions of the manufacturer. We used 100 ng of RNA for retrotranscription using NZY M-MuLV First-Strand cDNA synthesis kit (Nzytech, Caparica, Portugal). Real-time qPCR was carried out using PowerUp SYBR Green Master Mix (ThermoFisher, Waltham, USA), 1 μl of 1:10 cDNA dilution, and the primers shown in Supplementary Table S3. The reaction was performed using a total volume of 15 μl in a Step-One Plus Real-Time PCR system (ThermoFisher, Waltham, USA), cycling conditions were as follows: 50°C for 2 min, 95°C for 2 min, 40 cycles of 95°C for 15 s, and 30 s at 60°C; followed by a melting curve. To normalize the expression of the *KLF4, VDR, PPAR*γ, and *EDN1*, we followed the–ΔCT method using *YWHAZ* and *ALAS1* as reference genes.

### Chromatin Immunoprecipitation–qPCR

We used approximately one million HeLa cells per reaction. Chromatin shearing was performed using a sonicator (Branson). We carried out the ChIP using the ChIP Kit (Abcam, Cambridge, UK #ab500) following instructions of the manufacturer. For the pull-down step, the following antibodies and quantities were used: anti-Histone H3 antibody as positive control (Abcam, #ab1791; 2.5 μg), a Rabbit-anti-Mouse-Alexa Fluor 488 (ThermoFisher, Waltham, USA, #A-11,059; 5 μg), and an anti-PPARγ (Abcam, #ab59256; 5 μg). After DNA purification, we used 2 μl of DNA for qPCR, we carried out the reaction with the EDN1prom primers from Supplementary Table S3 using Power-Up SYBR Green Master Mix (ThermoFisher, Waltham, USA). For data analysis, we used the input percent method [100 × 2^(InputCT−CT(IP)^].

### Immunofluorescence

We cultured PAEC cells in μ-Slide 8-well chambers (ibidi, Gräfelfing, Germany) and performed the KD experiments by adjusting the volumes. After KD, cells were washed three times in PBS before being fixed with 4% formalin for 10 min at 37°C. After washing the cells six times in PBS, we proceeded to permeabilize them in PBS + BSA 1% (w/v) containing 0.1% Triton X (v/v). Then, we blocked them in PBS + BSA 2% (blocking buffer) for 1 h at room temperature (RT). We incubated the cells with the primary antibodies overnight in blocking buffer, washed three times with blocking buffer for 5 min, and incubated with the secondary antibodies and 4′,6-diamidino-2-phenylindole (DAPI; 1 μg/ml) in blocking buffer for 1 h in the dark. Finally, we washed the chambers three times for 5 min in PBS and mounted them in ProLong Diamond Antifade Mountant (ThermoFisher, Waltham, USA). Images were acquired using a Leica DMI6000 inverted microscope with an integrated confocal module SP5 (Leica Microsystems, Germany). The settings used for confocal imaging were maintained in the samples

The following antibodies and dilutions were used are as follows: anti-ET-1 (Abcam, Cambridge, UK, #ab2786, 1:500), phalloidin-Alexa488 (Abcam, #ab22744, 1:1,000), and Alexa Fluor 594-conjugated goat anti-mouse (ThermoFisher, Waltham, USA, #A-11005, 1:1,000).

### Image Analysis

All the images were processed with ImageJ (v.1.8.0). We did two experiments for HeLa and two for PAECs, in each we had duplicates for each treatment and we imaged two different places per well. We then selected between 15 and 30 whole cells from each image and quantified the mean fluorescence for each of them on the ET-1 channel. We subtracted the background for each photo. Settings were maintained in the same ibidi chambers, and all treatments were relativized to the Mocks within their slide.

### ET-1 Quantification Cell Media

We plated PAECs in passages 2–5 in 12-well plates, at 80–90% confluence we transfected them with the previously described conditions. Other experimental conditions tested were with DMSO (1%), the agonist of PPARγ Rosiglitazone (RGZ, 10 μM) and a PPARγ antagonist GW6992 (10 μM). We had a total of four biological replicates. After extracting the media, we centrifuged it at 14,000 g for 5 min to get rid of cellular debris. Moreover, we extracted protein from each well to correct ELISA results with the total protein present in the cells.

We quantified ET-1 levels using the Endothelin-1 Quantikine ELISA kit (R&D Systems, Minneapolis, USA). First we diluted the media 1/125 in the appropriate buffer and used 75 μl of the dilution for the assay. We incubated the media with 200 μl of assay buffer for 1 h at RT in a shaker. Then we washed the plate and incubated it with the anti-ET-1 conjugated antibody for 3 h at RT in a shaker. Finally, we washed the plate and added the substrate. We incubated it for 30 min before adding the stop solution and reading the results in an iMark microplate absorbance reader (BioRad, Hercules, USA).

### Statistical Analyses

For every experiment, we first performed a Shapiro test to assess normality. Then, we proceeded to analyze the data using a Kruskal-Wallis with Dunn's correction for multiple comparisons or Student's *t*-test for paired samples. Data were analyzed using R and plotting was performed with the ggplot 2^15^ and the ggpubr packages. Comparisons were considered statistically significant when *p* > 0.05, for multiple comparisons we used the adjusted *p*.

## Results

### Common Variation Can Be Found in *EDN1* Regulatory Regions

**Figure 1:**
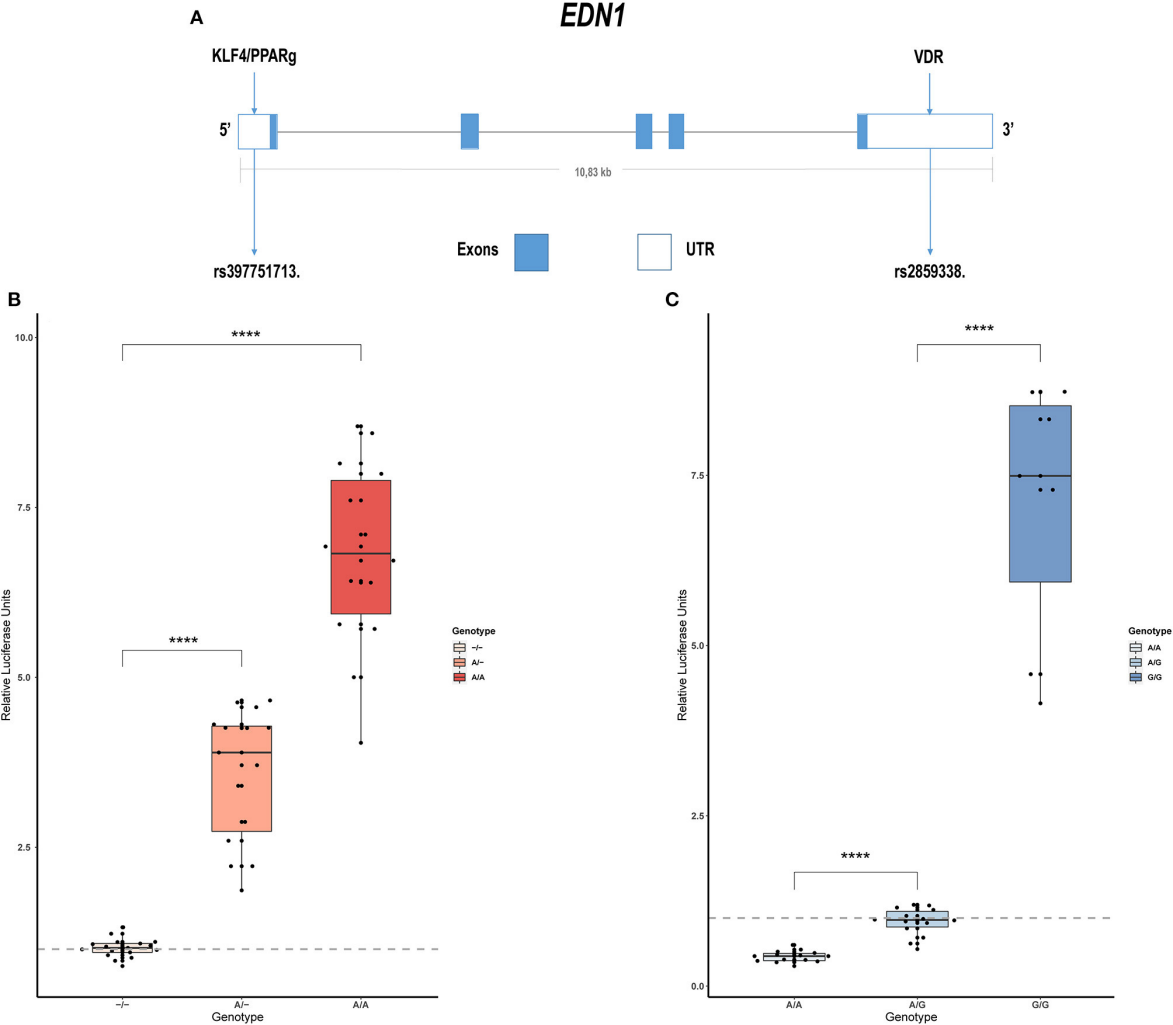
Common variants in EDN1 regulatory regions show different activities depending on the genotype. **(A)** Schematic view of the EDN1 gene with the location of the SNPs and the predicted transcription factors to interact with them. **(B)** Comparison of the different genotypes of rs397751713 show increased activity in the A/A genotype and A/- when compared to the commonest genotype –/– (*n* = 6). **(C)** Comparison of the different genotypes of rs2859338 show increased G/G genotype activity and reduced activity in A/A compared with the commonest genotype A/G (*n* = 4). Data are represented as box plots depicting the quartiles and dots representing technical replicates. A Kruskal-Wallis test with a Dunn's correction was used for statistical analysis (****p > 0.0001). EDN1, Endothelin-1.

Sanger sequencing of *EDN1* was carried out in 21 patients with IPAH and 15 with APAH. Variants were only found in the regulatory regions of the gene, not in the coding region, which is highly conserved. In the 5' UTR we found c.-131delA (rs397751713; Figure 1A) and in the 3' regulatory region g.12298751G >A (rs2859338; Figure 1A). Both of them are common single nucleotide polymorphisms (SNPs) in the European population and are classified as Benign in Varsome. Although we found slight differences in the genotype frequencies between our cohort and the Ensembl database (Table 1), they did not meet statistical significance. After a closer *in silico* analysis, we decided to carry out functional assays.

**Table 1:** Frequencies of the SNPs detected in *EDN1* UTRs in our cohort and ensemble.

**Genotype**	**Patients**	**Frequency**	**Ensembl**
**rs397751713**			
A/A	3	0.08	0.112
A/-	10	0.28	0.364
-/-	23	0.64	0.523
**rs2859338**
G/G	10	0.30	0.355
G/A	20	0.61	0.514
A/A	3	0.09	0.131

*SNPs, single nucleotide polymorphisms; UTR, untranslated region; EDN1, Endothelin-1*.

### The Common SNPs Are Predicted to Lower the Affinity of Some TFs

We used the bioinformatics software Genomatix to predict the TFs binding in the whole 5' UTR and 3' regulatory region. Among the 65 possible candidates provided by the Genomatix software, those with binding sites that included the SNPs detected were selected. This way, the candidates were reduced to KLF4, PPARγ, nuclear hepatocyte factor 4α (HNF4A) for 5' UTR and VDR in the 3' position.

Within these four candidates, we were only able to establish a nexus with PAH in three cases: KLF4, PPARγ, and VDR, with values for its similarity matrix of 0.961, 0.842, and 0.974, respectively.

### Promoters Carrying the Different *EDN1* c.-131del (rs397751713) Genotypes Show Differential Activity *in vitro*

We cloned into the p.GL3 luciferase reporter vector *EDN1* promoters carrying the different genotypes of the common SNP rs397751713. We found that the most common form c.-131del in homozygosity shows lower luciferase expression (–/–; 1.02 ± 0.14) when compared with the less common ancestral adenine insertion, both in heterozygosity (A/–; 3.58 ± 0.98; *p* > 0.0001) and homozygosity (A/A; 6.79 ± 1.36; *p* > 0.0001; Figure 1B).

### The 3' Regulatory Region Variant g.12298751G>A (rs2859338) Genotypes Show Different Activity *in vitro*

In the same manner, we tested the effect of the different genotypes of rs2859338 using the p.mirGLO luciferase reporter vector as this SNP is located in the 3' of *EDN1*. In this case, we used the most common heterozygous form to normalize and compare (A/G; 0.94 ± 0.2). The homozygous genotype A/A showed less expression than the most common variant (A/A; 0.43 ± 0.08; *p* > 0.0001), while the less common A >G substitution in homozygosity showed a high increase in luciferase expression (G/G; 7.04 ± 1.91; *p* > 0.0001; Figure 1C).

### The KD of KLF4, PPARγ, and VDR Increases *EDN1* MRNA Levels in PAECs

**Figure 2:**
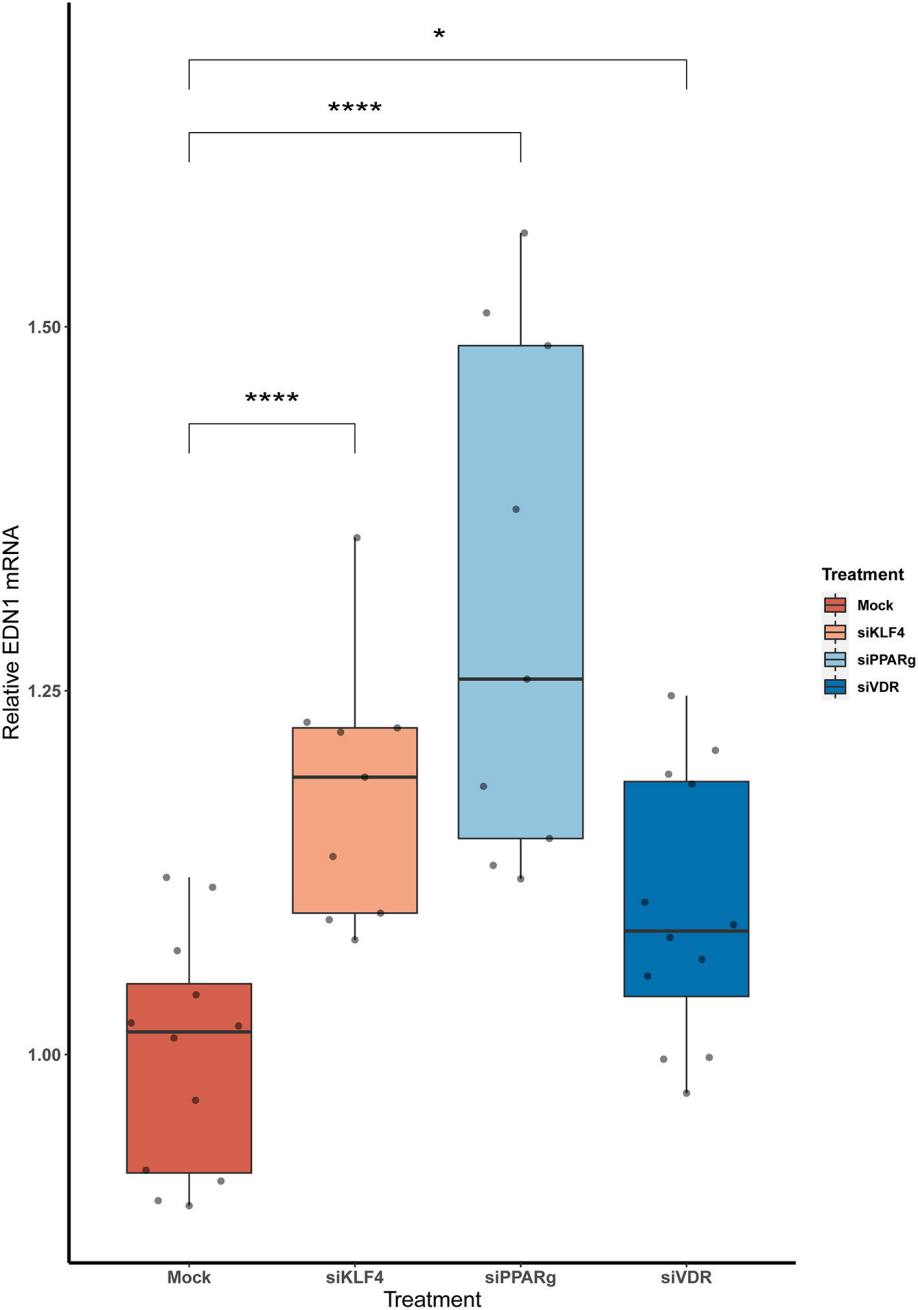
The KD of PPARγ, KLF4, and VDR increase the mRNA levels of EDN1. Data are shown as box plots depicting the quartiles (*n* = 3). Dots represent technical replicates. A Kruskal-Wallis test with a Dunn's correction was used for statistical analysis (*p > 0.05, ****p > 0.0001). EDN1, Endothelin-1; PPARγ, peroxisome proliferator-activated receptor γ; KLF4, Krüppel-Like Factor 4; VDR, vitamin D receptor.

We measured *EDN1* mRNA expression 48 h after the KD of the KLF4, PPARγ, and VDR. We found that the overall *EDN1* levels were increased in all of them when compared to the Mock siRNA (Figure 2). siPPARγ showed the greatest increase (1.31 ± 0.17, *p* > 0.0001), followed by siKLF4 (1.18 ± 0.08, *p* > 0.001) and then siVDR (1.1 ± 0.09, *p* > 0.02).

### PPARγ Binds to the EDN1 Promoter and Is Influenced by the A/A Genotype of rs397751713

**Figure 3:**
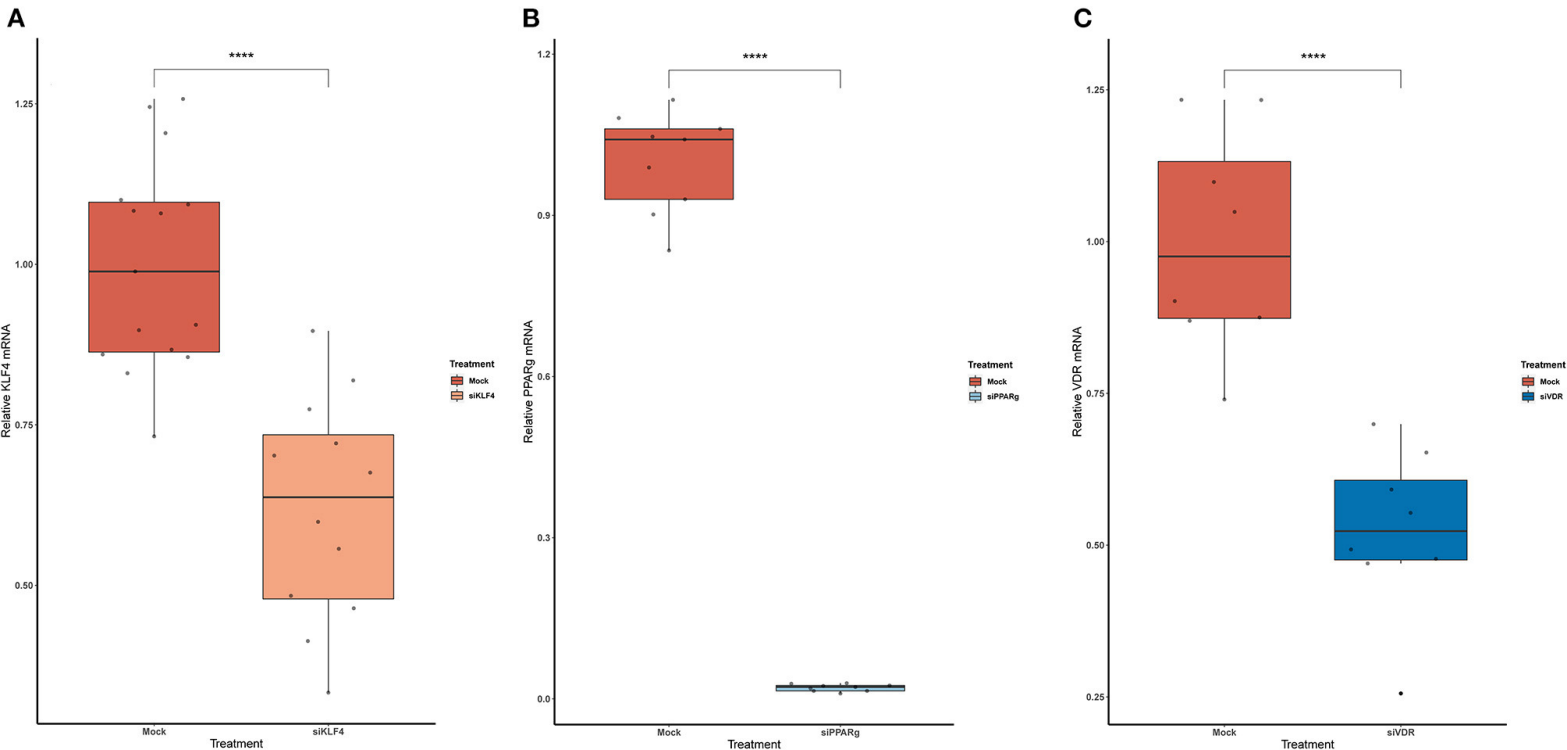
Knock-Down efficiency for KLF4 **(A)**, PPARg **(B)**, and VDR **(C)**. Data are shown as box plots representing median ± quartiles (*n* = 4). Dots represent technical replicates. A Student's *t*-test was used for statistical analysis (****p > 0.0001). PPARg, peroxisome proliferator-activated receptor g; KLF4, Krüppel-Like Factor 4; VDR, vitamin D receptor.

To test if rs397751713 genotypes influenced the binding of KLF4 or PPARγ, we did a KD of KLF4 using siRNA. We first optimized the reaction in primary PAECs getting around a 38% inhibition for KLF4 (Figure 3A), while for PPARγ it went up to 98% (Figure 3B), in both cases we used the maximum recommended siRNA amount.

**Figure 4:**
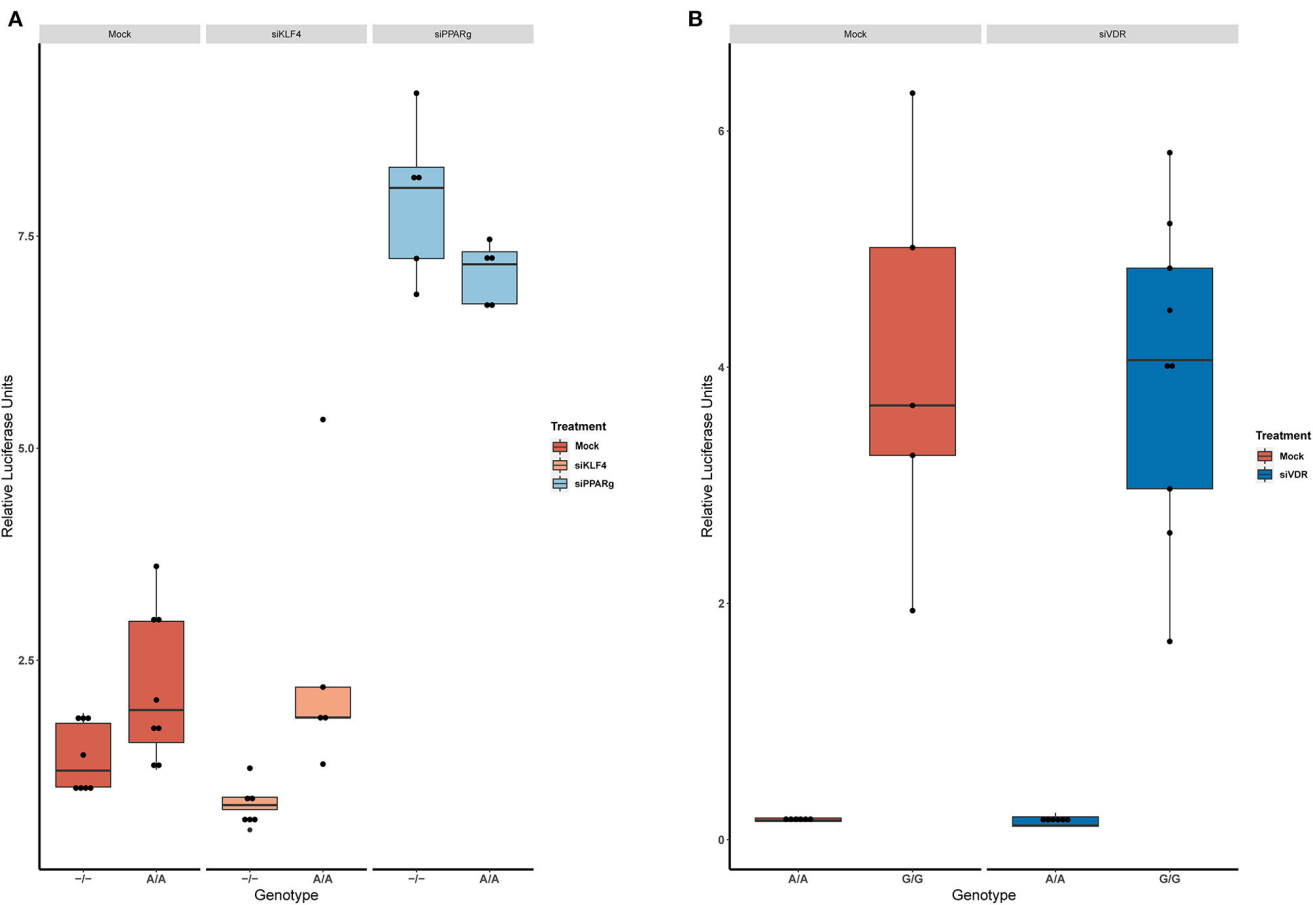
KD experiments demonstrate that PPARγ binds in rs397751713. **(A)** Comparison of the luciferase activity in the homozygous genotypes of rs397751713 after different treatments, siKLF4 and Mock show the same pattern while siPPARγ increases the activity of both genotypes to similar levels (*n* = 3). **(B)** Comparison of the luciferase activity in the homozygous genotypes of rs2859338 after Mock or siVDR treatment shows the same pattern. Data are shown as box plots representing median ± quartiles. Dots represent biological replicates. PPARγ, peroxisome proliferator-activated receptor γ.

**Figure 5:**
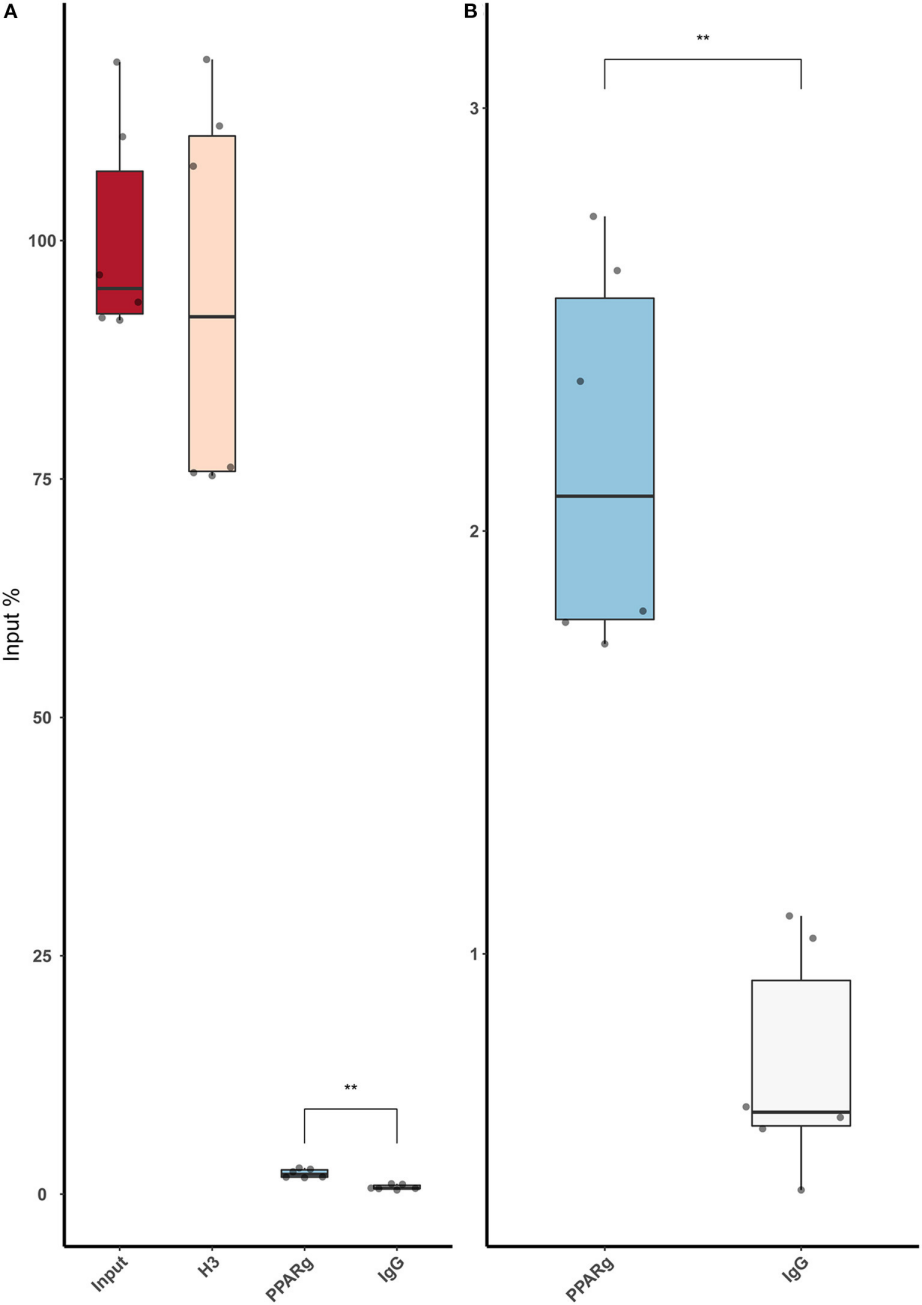
ChIP-qPCR using an anti-PPARγ antibody demonstrates that low amounts of PPARγ binds around rs397751713. **(A)** Full plot with the input chromatin used in the assay, a positive control (H3), and a Rabbit IgG. **(B)** Comparison between the control IgG and PPARγ amplification. Data are shown as box plots representing median ± quartiles. Dots represent technical replicates from two biological replicates. A Student's *t*-test was used for statistical analysis (**p > 0.01). PPARγ, peroxisome proliferator-activated receptor γ.

Then, we replicated the conditions in HeLa and transfected the p.GL3-EDN1prom with the homozygous genotypes (–/– and A/A). The results showed the same pattern between mock and siKLF4 (Figure 4A). However, siPPARγ showed a completely different pattern compared to control (Figure 3A), increasing luciferase activity to similar levels between –/– and A/A (7.96 ± 0.93 vs. 7.06 ± 0.35). This indicates that PPARγ binds in this position and its affinity could be reduced by the A/A genotype. Moreover, after pulling-down PPARγ, we were able to amplify this region in ChIP-qPCR assay (Figure 5). Meaning that PPARγ binds inside the 70 bp region we amplified, as it showed a greater input % (2.17 ± 0.45) than the negative control IgG (0.71 ± 0.26; Figure 5B).

In the case of rs2859338, we silenced VDR to test if it was bound in our target region. After optimization, we managed to lower VDR expression by 48% (Figure 3C). The luciferase results showed the same pattern for both genotypes treated with Mock (A/A 0.16 ± 0.01; G/G 4.04 ± 1.68) and siVDR (A/A 0.15 ± 0.05; G/G 3.96 ± 1.33; Figure 4B).

### PPARγ KD Increases ET-1 Production in HeLa and PAECs, While KLF4 Does It in HeLa and VDR in PAECs

**Figure 6:**
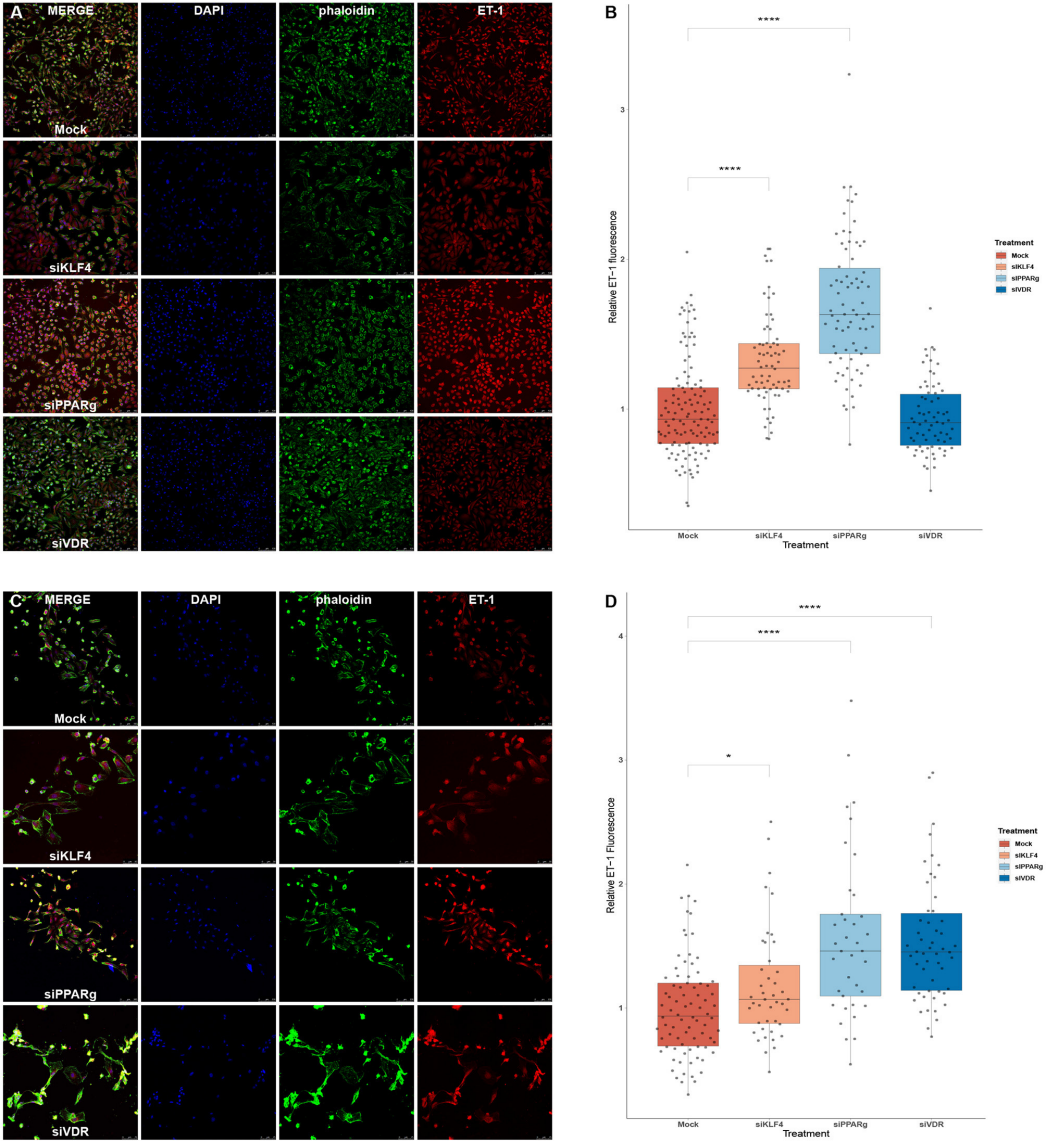
Silencing PPARγ increases ET-1 production in HeLa and PAECs while VDR does only in PAECs and KLF4 in HeLa. **(A)** Representative immunofluorescences for HeLa cells. **(B)** Quantification of ET-1 production using immunofluorescence in HeLa, treatment with siKLF4 and siPPARγ increases ET-1 fluorescence (*n* = 4). **(C)** Representative immunofluorescences for PAECs. **(D)** Quantification of ET-1 production using immunofluorescence in PAECs, treatment with siPPARγ and siVDR show the highest increase in ET-1 fluorescence, while siKLF4 has a slight increase (*n* = 6). Data are represented as box plots depicting the quartiles and dots representing single cells measured. A Kruskal-Wallis test with a Dunn's correction was used for statistical analysis (*p > 0.05, ****p > 0.0001). PPARγ, peroxisome proliferator-activated receptor γ.

We used immunofluorescence to confirm that the increase in mRNA levels led to higher amounts of ET-1 production in PAECs. We first experimented in HeLa cells to see if a greater KD efficiency for VDR and KLF4 changed the results. HeLa cells showed increase ET-1 levels after the treatment with siKLF4 (1.31 ± 0.32, *p* > 0.0001) and siPPARγ (1.68 ± 0.43, *p* > 0.0001) while siVDR had more or less the same as WT (0.94 ± 0.23, n.s.; Figures 6A,B). In PAECs, siPPARγ showed the highest increase in ET-1 (1.57 ± 0.66, *p* > 0.0001) followed by siVDR (1.55 ± 0.53, *p* > 0.0001), siKLF4 had a slight increase barely significant (1.17 ± 0.45, *p* > 0.031; Figures 6C,D).

### ET-1 Levels in Media Are Not Increased After Silencing

**Figure 7:**
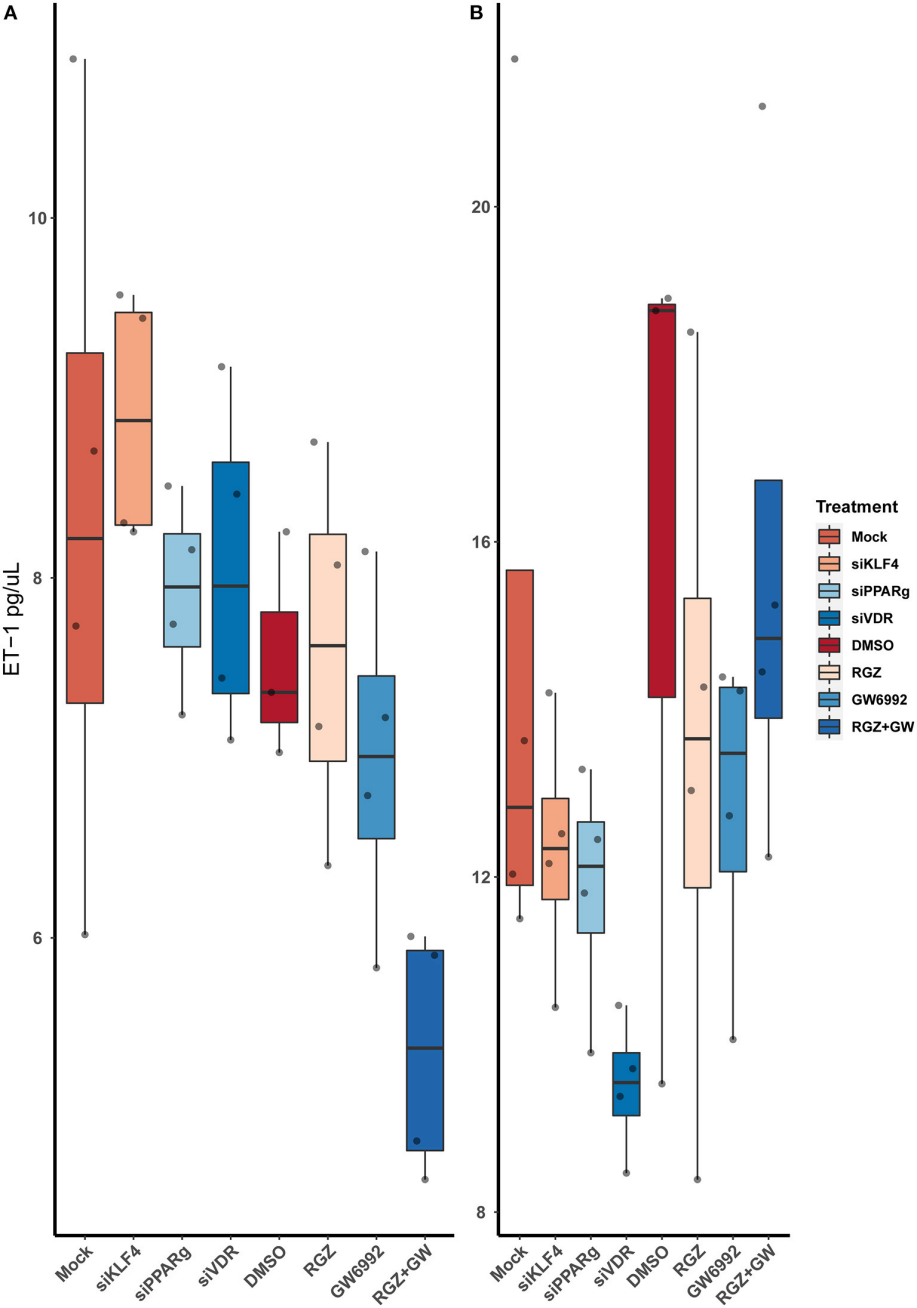
Quantification of ET-1 by sandwich ELISA. **(A)** Raw values. **(B)** After normalizing against the total protein from the well. Data are shown as box plots representing median ± quartiles. Dots represent biological replicates (*n* = 4). A Kruskal-Wallis test with a Dunn's correction was used for statistical analysis.

After running an ELISA against ET-1 using cell-culture media, we did not find any statistically significant difference between the treatments. Moreover, attenuating or activating PPARγ did not change the overall levels of ET-1 in the media (Figure 7).

## Discussion

Genetic screening in PAH is usually performed by limited gene panels or whole-exome sequencing that neglect non-coding regions. Regulatory regions as promoters, UTRs, and other regulatory regions have been historically understudied.

In this study, we analyzed *EDN1* regulatory regions in the search for variants that could modulate *EDN1* expression, and then we coupled functional studies with patients' data to dissect the effect of these changes in a translational way.

In our screening, we only found three variants, the two common SNPs rs397751713 and rs2859338 in regulatory regions, and the already reported p.Lys198Asn within the coding sequence (13). The luciferase assays we performed for these non-coding variants showed a marked increase in the luciferase activity for the less frequent homozygous genotype of both SNPs. Looking at this data from an evolutionary perspective, we realized that the less frequent genotypes of both these SNPs were the ancestral forms, still present in the oldest clades. Besides, ET-1 is a very well-conserved and potent vasoconstrictor (16).

Using *in silico* tools, we predicted that KLF4/PPARγ could bind where rs397751713 is located, while VDR could bind to in rs2859338. We determined that silencing PPARγ increased the luciferase levels in all the tested genotypes, while the silencing of KLF4 showed the same pattern as the Mock. This suggests that PPARγ bind at this position, and at least in our static conditions KLF4 is not. PPARγ seems to interact with both genotypes, so the expression differences between the genotypes could be a matter of affinity, as PPARγ may be able to bind more efficiently to –/– genotype leading to an inhibition of the *EDN1* expression, while the Adenine insertion would have less PPARγ binding affinity and thus, a higher expression of *EDN1*. However, silencing VDR showed the same results as in the basal luciferase assay, meaning that VDR cannot bind at rs2859338.

To evaluate the influence that these TFs play on ET-1 levels, we performed KD experiments in PAECs. We first measured *EDN1* mRNA and found increased expression in all siRNA treatments. The result was almost non-significant in the case of siKLF4 with barely a 10% increase, while siVDR showed an 18% increase, and siPPARγ a 31%. Using the same conditions, we used immunofluorescence to measure ET-1 protein production at a cellular level, first in HeLa, and then in PAECs. Both of these cell lines showed increased ET-1 levels of more than 50% after silencing PPARγ. While silencing KLF4 was more effective in HeLa (31%) than it did in PAECs (17%), however, VDR resulted in increased ET-1 production in PAECs (55%). Altogether, in a static culture, PPARγ probably plays a bigger role in regulating ET-1 than KLF4, as its expression is triggered by shear stress (17), or VDR does, as maybe their influence could be played indirectly.

Krüppel-Like Factor 4 is a well-known TF in the PAH context. It is expressed in the vascular endothelium, promoting anti-inflammatory and anticoagulant states. The lack of this TF in the vascular endothelium was shown to exacerbate hypoxia-induced PAH and increase the expression of ET-1 (18). Our results support this, and recent ChIP-seq data show that it binds to the *EDN1* promoter in the position we studied (17).

Besides, PPARγ influence in ET-1-mediated vascular damage is well-known (19), but how this interaction happened has not been shown until now. PPARγ is a ligand-dependent TF, which binds to hormonal response elements in promoters of target genes, mainly related to adipogenesis and secondarily to glucose metabolism (20). In PAH, remodeled and muscularized precapillary arterioles show frequently reduced PPARγ expression in endothelial cells. PPARγ not only regulates hypoxia-induced ET-1 levels but also other components of the ET-1 signaling pathway, such as Endothelin-Converting Enzyme 1 (ECE-1) mRNA levels, ETA, and ETB (21). Due to the above, it is believed that PPARγ agonists could reverse pulmonary vascular remodeling (22, 23). Furthermore, a malfunction of BMPR2 has been shown to decrease endogenous PPARγ activity and promote metabolic pathways associated with vascular remodeling (24). Experiments in animal models support the results obtained in this work (19). Furthermore, it appears that there is a reciprocal regulation between Endothelin and PPARγ (25).

The notion that several extrapulmonary organs (heart, skeletal muscle, and adipose tissue) show vascular and metabolic abnormalities suggests that PAH is a systemic rather than exclusively pulmonary disease. Dyslipidemia and insulin resistance are evident in animal models of PAH and human disease (26). In fact, many drugs that act as ligands for the PPARγ receptor are currently used as a treatment for type 2 diabetes (21).

The VDR is a TF that is activated in the presence of calcitriol, the active form of vitamin D. The relationship of VDR with genes involved in the regulation of the vascular tone, such as ET-1 (vasoconstrictor) and nitric oxide (NO, vasodilator), has been demonstrated. The regulation of NO by the VDR is direct, but it does not appear to be so in the case of ET-1. Previous reports showed that the regulation of ET-1 by VDR would not alter the expression of preproendothelin, but would act on the ECE1, generating more active endothelin (27). However, VDR increases the amount of NO by directly activating endothelial NOS (eNOS), generating a vasodilator effect (27). So it would be expected that the system would saturate rapidly in the case of ET-1, as the amount of preproendothelin mRNA did not increase (27), and the overall result would be vasodilation. This hypothesis is supported by a recent report by Callejo et al. (28) and our results. As after silencing *VDR*, ET-1 expression increased *in vitro*.

ELISA technique demonstrated that even though ET-1 production is increased at the cell level, it does not increase the levels of ET-1 secreted to cell media. None of the treatments tested showed any relevant difference when compared with the controls, and even pharmacological inhibition or activation of PPARγ had no effect on the secretion of ET-1. This leads us to think that ET-1 secretion may be tightly regulated and could be stopped after reaching a certain level of ET-1 in the media, it could also be recaptured by Endothelin B receptor (ETBR) in the ECs, or it could be affected by the absence of the Smooth Muscle Cells (SMCs) that would usually be the target of this peptide. Our fluorescence data show the accumulation of vesicles around the nuclei, but not near the membrane to be secreted (29), which makes sense with what we detected in the ELISA.

Pulmonary Arterial Hypertension is a complex disease where genetic information is limited to the main causal genes and little is known about the role of common variation (30, 31). Over the last decades, the capability of detecting genetic variation has increased; we can now try to use common variation to explain phenotypic expressivity. Common variants influencing the regulation of the ET-1 pathway had been proposed previously when it was shown that the response of patients with PAH to ERAs could be modified by a common intronic SNP in the GNG2 gene (32). But in that study, no SNP in EDN1 was statistically significant or studied in-depth, and the molecular effect of the GNG2 SNP was not analyzed *in vitro*. Another example in a closely related pathway is the angiotensin II type 1 receptor (AGTR1), where patients harboring a homozygous C/C allele for rs5186 showed a later age of diagnosis (33, 34). Also, an SNP (rs12483377) in the endostatin gene (Col18a1) showed different serum levels in patients that were homozygous for p.Asp1675Asn instead of the ancestral Asparagine, while the levels of endostatin impacted survival (35). But common variants influencing PAH not only appear in cardiovascular-related pathways, an SNP in *Sirtuin 3* (*SIRT3*; rs11246020) was associated with IPAH, as it lowered SIRT3 activity a 30% favoring glycolysis in mitochondria (36). The latest additions to this list are an SNP in an enhancer locus near SOX17 (rs10103692) that was associated with PAH, and a variant in *HLA-DPA1/DPB1* (rs2856830) that was associated with I/HPAH and showed different survival depending on the genotype (CC vs. TT) (37).

The mutational load could modulate gene expression and alter patients' phenotypes. PAH variability could be explained by how common variation influences the different pathways involved in the pathogenesis, it could be a way to explain how mutations with the same effect can have very different phenotypes. The next step should be screening the SNPs we identified in patients undertaking ERAs, as they could explain the very different responses to this treatment. We would like to encourage the biggest cohorts to go beyond rare variation and test in detail possible genetic modulators.

The main limitations of this study are that due to the small size of our cohort, we cannot draw conclusions at the different outcome levels. Moreover, we have been working with plasmids constructed with limited fragments of the regulatory regions of *EDN1* in cell lines. The results on KLF4 and VDR could need a better silencing efficiency to show in the luciferase assay, our confidence in them is based on repetition, and we can see the same patterns at mRNA and protein levels. Besides, culturing PAECs in physiological conditions could have changed some of our results.

In conclusion, we show how common variants in *EDN1* regulatory regions could alter ET-1 levels. We validated that PPARγ binds in rs397751713 and heavily influences ET-1 regulation *in vitro*. Furthermore, KLF4 and VDR influence ET-1 production in a cell-dependent manner.

## Data Availability Statement

The raw data supporting the conclusions of this article will be made available by the authors, without undue reservation.

## Ethics Statement

The studies involving human participants were reviewed and approved by Comité Ético de Investigación Clínica de Galicia. The patients/participants provided their written informed consent to participate in this study.

## Author Contributions

ML-D, CS, and DV participated in conceptualization and writing—original draft preparation. ML-D, CS, and LM-M participated in *in vitro* experiments and data analysis. DV participated in project administration and funding acquisition. AB participated in samples acquisition. All authors have read and agreed to the published version of the manuscript.

## Funding

This work was funded by the Cardiovascular Research Network of Instituto de Salud Carlos III de Madrid (RD06/0003/0012) Spanish Ministry of Science and Innovation PI18/01233 and Janssen Pharmaceuticals. CINBIO has financial support from Xunta de Galicia and the European Union (European Regional Development Fund-ERDF) (PO FEDER ED431G/02). ML-D and LM-M are supported by a Xunta de Galicia predoctoral fellowship (ED481A-2018/304; IN606A-2020/006). CS is supported by a Ministerio de Universidades FPU predoctoral fellowship (FPU19/00175).

## Conflict of Interest

The authors declare that the research was conducted in the absence of any commercial or financial relationships that could be construed as a potential conflict of interest.

## Publisher's Note

All claims expressed in this article are solely those of the authors and do not necessarily represent those of their affiliated organizations, or those of the publisher, the editors and the reviewers. Any product that may be evaluated in this article, or claim that may be made by its manufacturer, is not guaranteed or endorsed by the publisher.
